# American Academy of Dental Science

**Published:** 1892-06

**Authors:** 

**Affiliations:** American Academy of Dental Science


### AMERICAN ACADEMY OF DENTAL SCIENCE.

THE regular monthly meeting of the American Academy of Dental Science was held at the Boston Medical Library Association rooms, on Wednesday, January 6, 1892, at 7.30 P.M., President Brackett in the chair.

Papers were read as follows: By Dr. H. F. Hamilton, subject: "Description of a Case of Regulating."

(For Dr. Hamilton's paper, see page 416.)

By Dr. E. G. Tucker, subject: "History of Dentists who have graduated under Drs. Harwood and Tucker."

(For Dr. Tucker's paper, see page 348.)

### DISCUSSION OF DR. HAMILTON'S PAPER.

*Dr. Meriam.*—I would like to ask Dr. Hamilton whether the wisdom tooth erupted?

*Dr. Hamilton.*—Not until several years after the regulation was completed.

*Dr. Meriam.*—Was there much movement in the lower jaw? Was it possible for the patient to throw his jaw much forward?

*Dr. Hamilton.*—I should think he had rather less than the average movement.

*Dr. Banfield.*—Was the plate changed from time to time?

*Dr. Hamilton.*—Yes, the plate had to be changed, because it was rapidly worn away by the closing of the teeth. In four months' time I made three or four plates. At first I intended to make metal plates, or to put on the rubber plates metal caps, but I found that rubber was easier to shut into, so I let the patient wear out one plate and then made another.

*Dr. Andrews.*—How deep were the depressions on the plate?

*Dr. Hamilton.*—About an eighth of an inch; deep enough to get a firm hold on the occluding teeth.

*Dr. Baker.*—I should like to ask Dr. Hamilton if both of these plates were worn at the same time.

*Dr. Hamilton.*—No; they were worn alternately.

*Dr. Clapp.*—I understood Dr. Hamilton to say that the second molars were not covered with the plate, and after they had come into position the patient could not shut the mouth in the old way. I would like to ask if, before the plates were taken off, the second molars had come down in the way he designed that they should?

*Dr. Hamilton.*—They came on a line with the others; they all came together.

*Dr. Clapp.*—It seems to me that is one of the practical points of the case,—allowing the second molars to come into position.

*Dr. Robinson.*—Could you not have jumped these teeth far enough forward at one jump?

*Dr. Hamilton.*—No; I should not think it advisable.

*Dr. Meriam.*—With regard to Dr. Tucker's paper, I want to say one word. I have said the same thing before, but I think perhaps it had better be in the presence of Dr. Tucker, and that is, that there must be among the archives of the Tuckers and the older dentists many of the earlier formulæ of dentistry, one of which he has referred to, and it seems to me that it is a good time to inaugurate a movement for the publishing of those formulæ, now that we have a journal that is willing to print interesting matter of this nature.

*Dr. Baker.*—Here is a tooth which is of interest; it was given to me by a lady about ten years ago, and she told me that it was filled by Dr. Joshua Tucker forty years before that (*i.e.*, 1840). The man from whose mouth the tooth came has been dead about ten years, and it is such an old affair, I thought I would bring it here this evening.

REMARKS BY DR. CLAPP UPON "COMBINATION FILLINGS."

*Dr. Clapp.*—The subject of combination fillings is one of great interest to me, and in looking over my records I find that the percentage of these in proportion to the whole number of fillings that I put in is very great. I believe that not one in ten realizes the practical utility of such fillings in the saving of teeth, and I doubt very much if one in twenty uses them to any great extent.

If the teeth are young or old, and of poor quality, to my mind it is much better to put in a combination filling of cement and gold than to fill entirely with gold. Of course, we have all been in the habit of lining up cavities with cements and filling them with gold afterwards. This is a practical and valuable method, and the principle may be extended very materially.

I have been in the habit for some time of putting cement into cavities, and, while the cement is soft, placing plastic gold right into it. By so doing, the gold of itself obtains the necessary retaining points; whereas, if you put the cement in and allow it to harden, then you must re-excavate and form undercuts to hold the gold. In crown cavities, for example, where the central portion of the tooth is largely decayed, but the overlapping enamel is still sufficiently strong to withstand the force of mastication, either amalgam or gold can be put in, and the surplus cement which is squeezed out by forcing the gold or amalgam into the cavity cut away. After waiting a minute or two for the cement to harden, the filling can be furnished with gold or amalgam, as the case may be. It seems to me that in many cases these fillings are better than if they were made of one material. We find oftentimes in connection with a large cavity on the mesial surface of a molar, a small pin-head cavity in the distal surface of the adjoining bicuspid. My experience is, that if I fill these smaller cavities with gold, decay will reappear, and the fillings tumble out.

In such cases I often use tin and gold, a combination which you have heard advocated by Dr. Hamilton and Dr. Briggs, and which was spoken of many years ago in an article by Dr. Abbott, of Berlin. It is very serviceable in the crown cavities of sixth-year molars, also in buccal cavities, and in the temporary teeth. The statement which has been made here by Dr. Hamilton and others, that these fillings after a time become hard, something like an amalgam filling, is a fact, which I have demonstrated many times in my own practice. One advantage of the combination of tin and gold is that it does not discolor the teeth, as amalgam. As to the use of amalgam and gold, I know that my combination of these materials lasts longer than the all-gold fillings which I formerly inserted in similar cases. Oftentimes, in talking with dentists, I ask them if they use this combination, and they will say "yes," and then remark, "Do you use a matrix?" Thus showing that they have no idea of the proper manner of putting in such fillings, and, consequently, no idea of their value in saving teeth. To correct such false ideas is the reason for my appearing before you to-night.

The use of cement and gold in the front teeth is, to me, a very important affair. Without first putting in cement in the case of large cavities, or moderately large cavities in the poorer quality of teeth, I feel that the chances for permanent success are small. Cavities in frail teeth, where it is difficult to obtain a good retaining shape, can be filled with gold and cement, and the cement will assist very materially in retaining the filling.

In large cavities, if we can have a considerable proportion of the filling cement, we have a better filling, as there is less metal, and the cement will adapt itself better to the tooth than any other material. Here are specimens showing the method of putting in these fillings. This one shows a large approximal cavity in a molar. If a matrix is adjusted and cement placed in the cavity, and, while it is soft, amalgam inserted, the cement will be crowded down next to the matrix, and, consequently, the edges of the cavity will not be completely protected by a metal. To overcome this, I put in the cement before putting on the matrix; then add a little amalgam, thoroughly incorporating it with the cement; then trim off around the cavity so that the edge towards the matrix is entirely exposed; then put on the matrix, and finish with amalgam or gold. In other cases I find it convenient to put on the matrix, and put a little amalgam next to it; then fill the body of the cavity with cement, and finish with amalgam or gold, or both, as the case may be. The large filling in the lateral incisor, which has been passed around for your inspection, and which, you will remember, is quite large,—restoring the contour of the distal portion of the tooth,—was made in the following manner: The cavity was prepared so that there was considerable undercut at the cervical wall; there was a very slight undercut towards the point. Cement was first put in; then plastic gold crowded into the cement; *but the gold did not go into any retaining point whatever.* After waiting a few minutes for the cement to become hard, the filling was finished with odd lots of gold that I picked up around the office, and with an extra hard blow of the automatic mallet. My idea was to see if the blow from the automatic mallet would break up the cement and prevent its being strong. Now, I want this filling tested. I may suffer a great disappointment to-night, because I don't know how strong the filling is, and it is possible that the gold may have no retention at all in the cement; but I do know that the mallet was put on very much harder than it would have been if the filling had been in the mouth, and the filling was very thoroughly finished with burnishes, plate-files, disks, and stones.

[The tooth was then given to the President, who removed the filling with strong excavators, and stated that, in his opinion, it was more firmly held in place than would have been possible in such a cavity without the use of cement.]

After seeing the pressure it required to break out this filling, I think I should dare trust it in the mouth. Of course, in filling these cavities, you are laboring under unfavorable conditions.

*Dr. Ainsworth.*—I would like to ask Dr. Clapp if the plastic gold was annealed before it was put into the cement.

*Dr. Clapp.*—If the bottle of plastic gold has been open any length of time, I usually anneal it; then put it into the cement, and crowd it in with a large instrument. A sufficient time must elapse for the cement to harden. After the cement has become a little hard, the plastic gold, inserted in one or more pieces, must be thoroughly consolidated with proper instruments. Then the filling can be finished as though no cement was there at all, using either plastic gold or foil.

The cement that I have used for five or six years,—perhaps even longer than that, up to within about six months,—has been Weston's. I have used it with a great deal of satisfaction; but, just before the summer vacation, it became so unmanageable that I had to give it up.

I then got some of Dawson's cement, and have used that a little since; but in my hands it is not satisfactory. I think it is a pretty strong cement, and for lining up cavities it will do very well. Years ago, before trying Weston's, I used Fletcher's porcelain cement, which, it seems to me, was the best I ever used. That, after a time, became cranky, the liquid becoming hard so quickly, after a fresh bottle was opened, that I gave it up. This autumn, after having had an unfavorable experience with Weston's cement, I wrote to Dr. Lewis, of Buffalo, who is the American agent for Fletcher's filling-materials, and asked him if he had Fletcher's porcelain cement, and if it was in good working order. In reply, he sent me a package of it, and I find that it works the same as it used to, and it seems to me to be a very good cement. Some of its qualities are these: You can mix it very thin for crown-work, etc., and it will set very hard. It is a rather slow-setting cement, and you can use it very nicely, even after it is quite hard. It then works like dry amalgam, and with an oil-burnisher takes on a fine polish.

*Dr. Mead.*—I would make a suggestion with regard to cements: the powder, when of long standing, absorbs moisture which somewhat impairs its virtues. It can be restored to its original condition by heating in a porcelain dish over a sand-bath.

*Dr. Clapp.*—I would like to speak of another thing in regard to plastic gold, and that is its great value in commencing fillings. I cannot resist giving you one demonstration of that fact. Here is a cavity on the buccal side of a molar, and you know sometimes these cavities are very sensitive, and it is difficult to get much of a retaining point. This cavity was prepared with an inverted cone-bur, which was put in and run right around in the cavity. There is no angle whatever in it. The walls are nearly perpendicular; the cavity is a little larger inside than out. The way I start such a case as this is to take a good-sized piece of plastic gold,—I use Steurer's usually for this purpose,—and with a large instrument pack it in, using a smaller instrument to carefully condense the gold about the edges. If the gold is fresh, I do not anneal it; otherwise I do.

*Dr. Ainsworth.*—Do you use copal ether or anything of that kind before starting the filling?

*Dr. Clapp.*—Sometimes, and, theoretically, it is the correct way, but I don't always do it.

*Dr. Ainsworth.*—Did you ever notice any leaking when copal varnish had been used?

*Dr. Clapp.*—If it is used very thin, so thin that the preparation looks like water, it doesn't seem to me that there can be any trouble from that.

*Dr. Parker.*—Have you ever used Williams's plastic gold?

*Dr. Clapp.*—Yes, but I have never found any gold that has the quality that Steurer's has. And the special quality that makes this gold valuable in such cases is that when you put an instrument onto it, it is like pushing your finger into a snow ball; it only condenses that which is directly before it; but if you put your finger into a ball of cotton, it pulls a great deal along with it. This gold is absolutely without fibre; it condenses before your instrument, like soft snow. I use plastic gold for commencing nine out of ten of the fillings that I put in. I don't care whether there is more waste or not; if I can save five minutes' time, that pays for the waste gold.

*Dr. Ainsworth.*—Do you ever commence with foil?

*Dr. Clapp.*—That depends on where the filling is, and how I can get at it. For the particular point in the cavity that I am at work upon I use that which I think will best serve the purpose. I use the various forms of gold interchangeably.

*Dr. Ainsworth.*—Without any hesitancy in regard to cohesion?

*Dr. Clapp.*—Yes, I have had no trouble whatever on that score.

*Dr. Eddy.*—Have you ever noticed that certain foils adhere more readily than others?

*Dr. Clapp.*—I have not thought of that particularly. I use, mainly, Morgan and Hastings's semi-cohesive foil.

*Dr. Potter.*—Would you consider it good practice to complete the filling with plastic gold?

*Dr. Clapp.*—Certainly.

*Dr. Payne.*—Do you find that plastic gold will burnish as well as cylinders or foil?

*Dr. Clapp.*—It burnishes, I find, exactly as well. I think it harder than foil when finished.

*Dr. Taft.*—The subject of combination fillings is always one of interest to me, and I feel under great obligations to Dr. Clapp for what he has shown us to-night, much of which I shall put into practice.

My experience with compound fillings began at the time Steurer's gold was introduced, and it has since that time been limited almost exclusively to amalgam and gold. Scarcely a day passes that I do not have occasion to insert one or more of these fillings, and I find them particularly valuable in large crown-cavities in molars, as well as in compound cavities when the matrix is used. After first making the cavity thoroughly antiseptic, my practice is to coat it with a very thin solution of sandarach varnish. By so doing the tubuli are closed, so that mercury is not expressed into them when force is applied to the amalgam, which should have as little surplus mercury as possible.

While I am not sure that compound fillings save teeth better than fillings made entirely with gold, I do know that this method saves time. In large crown-cavities in molars, for instance, where by other methods of insertion and finishing a filling will take an hour to perform, the time can easily be reduced by this method to ten or fifteen minutes.

My practice is to fill the cavity to within about an eighth of an inch of the top with amalgam; then carefully to work Steurer's gold into the amalgam, using broad points at first, then finer ones. I next use Watt's crystal gold, and finish the filling with foil or pellets. Thus we do away with all unnecessary undercuts, and reduce the time and fatigue of the operation to a minimum.

*Dr. Meriam.*—There is one filling which ought not to be lost sight of. It was introduced by a dentist of Brooklyn, N. Y., and consists of cement to which an alloy has been added. The durability of the cement is thus increased. Dr. Howe has given it as his opinion that there is something more than a mechanical union existing between the cement and alloy; there seems to be a chemical union, and the result is a greater resisting power against solution.

We have never failed in the mechanical work of filling; we can make our fillings stay in; we can make amalgam stick to gold, or gold stick to amalgam, and still be no further advanced than we were before, unless we impart to them a therapeutic action, or in some way prevent the edges of amalgam fillings from standing away from the teeth. With tin, on the other hand, which possesses therapeutic value, I have not known of a case where there has been this trouble. It is a material that can be nicely burnished, and is so soft that it adapts itself well to the edges and remains there; and while the tin itself may discolor, its stain is so little that it seems preferable to the union of gold and amalgam. The union between tin and gold may not be as strong as that between gold and amalgam, yet it is sufficiently strong for the purposes for which it is used.

*Dr. Banfield.*—Does Dr. Clapp have any preference in regard to the kind of amalgam he uses?

*Dr. Clapp.*—I have for five or six years used almost excusively Caulk's white alloy. It has proved itself better than any previously used. It works perfectly well as far as the union with gold is concerned; in fact, I have had no trouble in that respect with any amalgam.

In regard to the use of amalgam and gold in crown-cavities, as spoken of by Dr. Taft, I never use it in that way, for this reason: I consider cement, where it is entirely covered with gold, far preferable to amalgam. To me, there is an objection to a large amount of metal in a tooth; but the larger the core of cement the better the filling, to my mind. In large cavities, where there is an immense amount of amalgam and gold, there must, I believe, be some change of size due to thermal variations. This change in the size of a large metal filling may crack a frail tooth; therefore, I lay it down as a principle to put just as little metal as possible into a tooth, and still have enough for strength.

In regard to the amalgam creeping or crawling away from the edges, I have never seen it happen in the combination fillings of gold and amalgam such as I have described. Of course, I have seen many such cases where the filling was entirely amalgam. In one or two only have I seen decay reappear about my gold and amalgam fillings. I feel very much more confident of such fillings than I do of those made exclusively of either of these materials.

I thoroughly believe in using tin and gold. I often use these materials at the cervical margins in preference to amalgam.

*Dr. Banfield.*—I have used for a great many years what is called "Standard Alloy." It has given me better satisfaction than any other. I have recently seen some large fillings in molars extending from the crown to the approximal surface, and including some of the cusps. These fillings were made several years ago of "Standard Alloy," and are now in good condition; the edges perfect, and teeth well preserved.

*Dr. Taft.*—Since I have entirely discarded the use of silver alloys my combination fillings have been made with gold and copper amalgam. The result of my experience and observation with regard to silver alloy leads me to consider it the least desirable filling material that can be used.

*Dr. Banfield.*—Has Dr. Taft noticed any disintegration in his copper fillings? I understand that a number of dentists who have used copper amalgam have become disgusted with it, and have taken out all fillings made with that material.

*Dr. Taft.*—Occasionally I have found the disintegration mentioned, but it has almost always occurred in mouths where the secretions were of an acid nature, and where cement or gutta-percha fillings dissolve away very rapidly. The proportion, however, of cases where the fillings show disintegration or become cup-shaped is so small that I do not regard that objection as of any importance. If any of you have had occasion to take out copper-amalgam fillings, you will have noticed how much more dense they are than silver-amalgam fillings. Copper amalgam is used with very great advantage in cases of white decay, occurring on the buccal surfaces of molars. If such cavities are filled with copper amalgam, a hardening rather than a continued softening takes place. When the patient presents himself at a later day you do not find a leaky filling, as very often happens when a silver alloy has been used.

Almost all of the silver alloys, to a greater or less extent, have the disagreeable characteristic of bulging out or shrinking away from the margins of the cavity, and I have become thoroughly disgusted with them, and have entirely discarded their use.

*Dr. Banfield.*—As I understand it, white decay is found in mouths having an acid reaction. Are not these mouths, then, really the most unfavorable for copper amalgam?

*Dr. Taft.*—I certainly do not think so. If I had found such to be the case I should have discarded copper amalgam altogether. I can only speak from my own observations. Where copper-amalgam fillings show a cup-shaped appearance, I attribute it more to the fact that the amalgam has not been put in as dry as it should have been, or else that it has been put in under moisture, and a part of it has come away after the completion of the operation.

*Dr. Baker.*—I would like to ask Dr. Clapp if he uses his gold and amalgam fillings in cuspids,—say the distal surface, or the mesial surface of the first bicuspid?

*Dr Clapp.*—I often use it in the mesial surface of bicuspids. I don't remember that I have ever used it in a cuspid. If I use it in the mesial surface of a bicuspid, I put it in so that it will not show on the labial side, filling the whole of the buccal or labial wall with gold, so that there may be no discoloration. I have used to some extent a preparation, by Williams, of platinum and gold. It is a platinum foil on which gold has been deposited, and I oftentimes put a piece of this against the wall, and then pack amalgam against it. I have not, however, used it sufficiently to know whether it will be an absolute protection against discoloration. But, if amalgam is likely to show at the cervical portion of the cavity, I only put it part way, and fill the exposed portion with gold.

*Dr. Baker.*—As a matter of fact, then, amalgam invariably discolors, and the dentine is stained unless the cavity is lined with some other material.

*Dr. Clapp.*—All teeth are not discolored, but some are. I think the quality of the tooth has something to do with it; but it certainly discolors teeth to some extent, and for that reason one must use his ingenuity.

*Dr. Eames.*—For the past twelve years, since Dr. Kingsley spoke of using gold and amalgam together, I have followed his method, and have first filled with amalgam, and after it has hardened, removed a certain amount of it and completed the filling with gold. By this method I am able to control the shape of the filling as well as the amount of space which the amalgam is to occupy in the cavity. I am quite sure that when gold is placed in connection with amalgam, even though that has been in position two or three days, it controls subsequent changes in the amalgam. Such fillings do not crumble at the edges, or show the characteristic failures of pure amalgam. In very frail teeth I have allowed it to harden for the strength it would give, and then put in gold. At times, however, I use gold and amalgam, as Dr. Clapp has described; and join with others in thanking him for the admirable talk he has given us on this subject.

*Dr. Clapp.*—I have used copper amalgam a very few times. So far as I know, none of the fillings have dissolved out to any great extent; but they have become of such inky blackness, and the tooth itself has become permeated with the stain to an extent that I have never experienced in connection with the silver alloys.

*Dr. Taft.*—It is curious how experiences differ in regard to the use of different materials. Meeting Professor Putnam, of the Peabody Museum of Archæology, at Cambridge, one day, in the course of our conversation the subject of copper-amalgam fillings came up, and I remarked how much satisfaction and success I had in the use of it in the saving of teeth; and to what an extent it was being used by many others in our profession. He replied that he could very easily understand why this was so, as he had always found, in exhuming the bones or skeletons of the ancient mound-builders, "that wherever the bones had lain either in direct contact with or even near deposits of copper, they were in a perfect state of preservation." This shows that the salts of copper do have a very decided preservative effect on bone-tissue.

*Dr. Hamilton.*—Here are some little books that I have made for records, in connection with the ordinary examination-cards. They are of the same size as the cards, and have a page for each tooth. I put down special memoranda, and find it extremely useful in referring to the previous condition of the tooth in cases of special treatment.

*Dr. Banfield.*—I hold in my hand two mandrils, that are the best I have ever used for holding sand-paper disks.

They are made by the Claflin Dental Manufacturing Company, of Worcester. They have two points of advantage,—first, the screw-head and its adjacent parts are made very thin, allowing the disk to be carried far up between the teeth. Secondly, the screw-head is made so that only the circumference touches the disks, and by this means such a firm hold is obtained that there is no slipping when turned to the right.

WILLIAM H. POTTER, D.M.D.,
*Editor American Academy of Dental Science.*

